# The Impact of the Molecular Weight of Degradation Products with Silicon from Porous Chitosan–Siloxane Hybrids on Neuronal Cell Behavior

**DOI:** 10.3390/polym15153272

**Published:** 2023-08-01

**Authors:** Yuki Shirosaki, Federica Fregnan, Luisa Muratori, Saki Yasutomi, Stefano Geuna, Stefania Raimondo

**Affiliations:** 1 Faculty of Engineering, Kyushu Institute of Technology, 1-1 Sensui-cho, Tobata-ku, Kitakyushu 804-8550, Japan; 2 Department of Clinical and Biological Sciences and Cavalieri Ottolenghi Neuroscience Institute, University of Turin, Regione Gonzole 10, 10043 Orbassano, Italy; federica.fregnan@unito.it (F.F.); luisa.muratori@unito.it (L.M.); stefano.geuna@unito.it (S.G.); stefania.raimondo@unito.it (S.R.)

**Keywords:** nerve regeneration, organic–inorganic hybrids, chitosan, Si-containing degradation products

## Abstract

Silicon (Si) is an essential trace element in the human body and it exists in connective tissue as aqueous orthosilicic acid. Porous chitosan–3-glycidoxypropyltrimethoxysilane (GPTMS) hybrids can regenerate nerve tissue and recover sensor and motor functions. However, the structures and roles of the degradation products with Si extracted from the hybrids in nerve regeneration are not clear. In this study, we prepared porous chitosan–GPTMS hybrids with different amounts of GPTMS to amino groups of chitosan (chitosan:GPTMS = 1:0.5 and 1:1 molar ratios). The structures of the degradation products with Si from the hybrids were examined using time-of-flight mass spectrometry, and biological assessments were conducted in order to evaluate their potential use in the preparation of devices for nerve repair. Glial and motor cell lines and ex vivo explants of dorsal root ganglia were used in this study for evaluating their behavior in the presence of the different degradation products with Si. The structure of the degradation products with Si depended on the starting composition. The results showed that glial cell proliferation was lower in the medium with the higher-molecular-weight degradation products with Si. Moreover, motor cell line differentiation and the neurite outgrowth of dorsal root ganglion explants were improved with the lower-molecular-weight degradation products with Si. The results obtained could be useful for designing a new nerve regeneration scaffold including silicon components.

## 1. Introduction

Silicon is an essential trace element in the normal metabolism of higher animals and the formation of cartilage and connective tissue. In the 1970s, Carlisle et al. clarified the amount of silicon in tissue and investigated its roles in the human body [1,2,3,4]. Silicon is in human plasma as soluble silicic acid, usually at concentrations of approximately 0.5 mg/L. Carlisle et al. also discovered that silicon was localized in areas of active bone growth in mice and rats [2]. In the connective tissue of organs, such as ligaments, bones, skin, and aorta, 4–5 times more silicon has been observed compared to other tissues such as lungs, heart, and muscles [3,5].

Bioglass^®^, a SiO_2_-CaO-Na_2_O-P_2_O_5_ system, can promote osteoblast adhesion and proliferation, thereby helping bone formation [6,7,8,9]. The activated osteoblasts have many filamentous pseudopodia and microvilli on Bioglass^®^ surfaces. Insulin-like growth factor II (IGF-II) and extracellular matrix gene expressions were high when primary human osteoblasts were cultured in a solution using Bioglass^®^ extract. Additionally, eluates from Bioglass^®^, such as silicic acid and calcium, can increase apoptosis and promote DNA synthesis and mitosis in living cells. Aqueous orthosilicic acid is transported into the bone cells through a Na^+^–HCO_3_^−^ cotransporter, increasing collagen type I production, the gene expression of osteocalcin, and alkaline phosphatase (ALP) [10]. Based on the results of Bioglass^®^, the organic–inorganic hybrids, including siloxane units, have been investigated in bone regeneration. However, most discussions concluded that the Si ion concentration is dependent on bone regeneration such as osteoblastic proliferation and differentiation [11,12,13,14].

In previous studies, our group and collaborators have investigated the effect of siloxane units released from chitosan hybrids (e.g., membrane, porous scaffolds, hydrogels, and particles) with alkoxysilanes, such as GPTMS and tetraethoxysilane (TEOS). The siloxane units derived from chitosan–GPTMS at the appropriate concentration promoted cell proliferation and ALP activity [15], whereas those derived from chitosan–TEOS suppressed cell proliferation and promoted ALP activity, and those derived from chitosan–GPTMS–TEOS stimulated both cell proliferation and ALP activity of osteoblastic cells, even at the same Si concentration. As the amount of GPTMS increased, the number Si-O-Si bridging bonds in the hybrids increased. Additionally, the degradation products with Si from the chitosan–GPTMS hydrogels had a different effect on the osteoblastic cell viability, even at the same Si ion concentration [16]. These results suggest that the Si ion concentration in the medium and the structure of the degradation products with Si from the hybrids affect osteoblastic cell behavior. Considering the results of the hybrids with Si in the bone, we can also hypothesize a role in nerve regeneration. Therefore, when thinking of a possible application of this biomaterial in the preparation of devices for nerve repair, we investigated nerve tissue regeneration using chitosan–GPTMS hybrids [17]. Two hybrids, a solid membrane and a porous scaffold, were used for sciatic nerve regeneration and they showed different results. The solid membranes inhibited nerve regeneration; alternatively, the porous scaffolds improved nerve tissue regeneration, such as the number of fibers and axons, the sizes of the fibers and axons, and the myelin thickness. Nerve tissue regeneration led to recovered motor and sensory functions. We consider two reasons why the results were different. The first is that the interconnected porous structure of the hybrids (porosity > 90%, crosslinking degree = 35%) improved the permeability of oxygen, nutrients, and waste. The second is that the degradation products from the hybrids with Si were responsible for promoting nerve regeneration. Few papers have reported the effect of silica on nervous system regeneration. Parveen et al. reported that silica nanoparticles that accumulated in the corpus striatum of rat brains increased the formation of reactive oxygen species, depleted cellular antioxidants, and induced apoptosis [18]. Inoue et al. also showed similar results using silica nanoparticles for neuron cells [19]. However, the structures and roles of the species with Si in nervous system regeneration are not clear. Recently, Hattori et al. examined the structure and effect of the degradation products from the solid chitosan–siloxane hybrid membranes prepared with different alkoxysilane reagents, GPTMS, TEOS, and 3-glycidoxydimethoxymetylsilane (GPDMS) [20]. The structures of the degradation products with Si in the extractions were examined using time-of-flight mass (TOF-MS) spectrometry. Monomeric degradation products with Si detected in the extractions from chitosan–GPTMS and chitosan–GPDMS hybrids inhibited glial cell proliferation. Alternatively, the degradation products with Si from the chitosan–TEOS hybrid did not affect cell proliferation. The degradation products with Si were not taken up into the cell. The results indicate that the organic chains bonded to silicon atoms inhibited glial cell growth by adsorption to the cell surface or biological components such as proteins in the medium. To consider the correlation between in vivo results and in vitro results, the details of the extraction from porous chitosan–GPTMS should be investigated.

In this study, we focus on the degradation products, including silicon, in the extraction to analyze their structures and their effects on the nerve cells in vitro. We prepare porous chitosan–GPTMS hybrids with different amounts of GPTMS to amino groups of chitosan (chitosan:GPTMS = 1:0.5 and 1:1 molar ratio) that have shown good nerve regeneration [17]. The structure of the degradation products with Si from the hybrids is examined using TOF-MS spectrometry, and biological assessments are conducted using the RT4-D6P2T schwannoma cell line, a mouse motor neuron-like cell line (NSC-34), and an organotypic culture of rat dorsal root ganglion (DRG).

## 2. Materials and Methods

### 2.1. Preparation and Characterization of the Porous Hybrid Extractions

The porous hybrids were prepared using the freeze-drying method [17]. We used a planetary centrifuge (ARE-310, Thinky, Tokyo, Japan) at 2000 rpm (Program: MIX 5 min − DEFOAM 3 min) × 3, MIX 5 min) to yield the chitosan solution and mixtures with GPTMS.

The compositions were chitosan:GPTMS = 1:0.5 and 1:1 as the molar ratios, named ChG05 and ChG10, respectively. The obtained porous hybrids were cut into a square (10 mm × 10 mm, thickness = 2 mm) and sterilized with ethylene oxide gas (20% CAPOX, 45 °C, 50% humidity, Steri-Tech Inc., Saitama, Japan). Square specimens were soaked (0.3 mm^3^/mL) in ultrapure water (Life Technologies, Carlsbad, CA, USA) at 37 °C for 1 week. Extractions were filtered using a syringe filter (0.22 µm pore size) before the following analysis. The amount of Si(IV) and molecular weight of the degradation products in the extractions were measured using inductively coupled plasma emission spectrometry (ICP, ICPS-8000, Shimazu Corporation, Kyoto, Japan) and electron spray ionization TOF-MS spectrometry (JMS-T100LP, JEOL, Tokyo, Japan) [20]. The free amino groups in the extractions were measured using the ninhydrin method [21].

### 2.2. Biological Assessment

#### 2.2.1. Preparation of Culture Medium with Extracted Degradation Products

Square specimens were soaked (0.3 mm^3^/mL) in Dulbecco’s modified Eagle medium (DMEM, Sigma-Aldrich, St. Louis, MO, USA) at 37 °C for 1 week to obtain each concentration (0.05, 0.10, 0.50 mM) of Si(IV) culture medium. The culture medium was supplemented with 1% (4 mM) L-glutamine, 1% (0.1 mg/mL) streptomycin, and 100 U/mL penicillin (Sigma-Aldrich).

#### 2.2.2. RT4-D6P2T Cell Proliferation

In order to test for cell proliferation, RT4-D6P2T schwannoma cells (ATCC, CRL-2768) were cultured at 37 °C in a humidified atmosphere of 5% CO_2_ for 2, 4, and 6 days at an initial density of 0.3 × 10^4^ cells/cm^2^. The medium used contained Si and 5% fetal bovine serum (FBS; Invitrogen, Waltham, MA, USA). Cell proliferation was estimated using an MTT test [22]. The cultured cells were reacted with 5 mg/mL MTT for 4 h at 37 °C. The obtained formazan salts were then dissolved in dimethyl sulfoxide after the culture medium was taken out. A microplate reader (Asys UVM 340, Biochrom Ltd., Cambridge, UK) was used to measure the absorbance at 570 nm.

#### 2.2.3. RT4-D6P2T Total Protein Extraction and Western Blot

For the Western blot, RT4-D6P2T cells were also cultured for 2, 4, and 6 days at 37 °C in a humidified atmosphere of 5% CO_2_ at an initial density of 0.3 × 10^4^ cells/cm^2^ on glass slides. Cells were dissolved in boiling Laemmli buffer (2.5% sodium dodecyl sulfate, 0.125 M Tris-HCl pH 6.8) and then heated at 100 °C for 3 min to extract the total protein. A bicinchoninic acid assay kit (Sigma-Aldrich) was used to measure the protein concentration [23]. Equal amounts of proteins were loaded into each lane, separated by SDS-PAGE, transferred to a HybondTM C Extra membrane (Bio-Rad, Hercules, CA, USA), and blocked for 1 h at room temperature in 1 × TBST (150 mM NaCl, 10 mM Tris-HCl (pH 7.4), 0.1% Tween, and 5% nonfat milk (Bio-Rad). Membranes were incubated with primary antibodies diluted in TBST with 1% BSA (Sigma-Aldrich) overnight at 4 °C. Membranes were washed in TBST (4 times, for 5 min each) and then incubated with a peroxidase-linked secondary antibody (diluted in TBST with 1% BSA) for 1 h at room temperature. Using a ChemiDocTM Touch Imaging device (Bio-Rad), the enhanced chemiluminescence ECL technology was able to identify the specific binding. The list of primary and secondary antibodies is presented in Table 1.

#### 2.2.4. RT4-D6P2T Cell Morphology

RT4-D6P2T cells were seeded under the same conditions as the Western blot analysis in order to observe cell morphology. Cells were washed with PBS (Thermo Fisher Scientific, Waltham, MA, USA) and fixed with a 4% paraformaldehyde solution (Sigma-Aldrich) at each experimental time point. Fixed cells were permeabilized for 1 h at room temperature using PBS 0.1% Triton X-100. TRITC-conjugated phalloidin, diluted at 1:1000 in a blocking solution, was used to identify F-actin. This was achieved by incubating the sample for 1 h at room temperature, followed by three washes lasting 5 min each, and mounting it using a Dako fluorescent mounting medium (Dako GmbH, Glostrup, Denmark). A Zeiss LSM800 confocal laser microscope (Zeiss, Jena, Germany) was used to capture the images. Each sample was photographed in thirty different fields, and the number of cells was estimated using the ImageJ v1.48 program from the National Institutes of Health in Bethesda, Maryland, USA. The actin cytoskeleton organization was used to undertake a quantitative analysis of the cell morphology. Each cell received an arbitrary score that was expressed as the proportion of cells with poor, medium, or high actin cytoskeleton organization [24]. The development of lamellipodia and filopodia was also assessed. The percentage of cells with only lamellipodia or cells with lamellipodia plus filopodia was used to express the value.

#### 2.2.5. NSC-34 Cell Differentiation

Mouse motor neuron-like cells, NSC-34 (Cedarlane, CLU140) [25], were cultured at 37 °C in a humidified atmosphere of 5% CO_2_ and seeded at an initial density of 0.3 × 10^4^ cells/cm^2^ on glass slides in a medium with the degradation products with Si extracted from the porous hybrids. After 2 days of culture, the medium was changed to DMEM-F12 (Sigma-Aldrich) with the differentiation products with Si extracted from the porous hybrids supplemented with 1% FBS, 1% (4 mM) L-glutamine, 1% (0.1 mg/mL) streptomycin, 100 U/mL penicillin, and 1 µM retinoic acid (Sigma-Aldrich) and cultured for 5 days. The cultured cells were rinsed with PBS and then fixed in a 4% paraformaldehyde solution for immunofluorescence examination.

Before being incubated with the primary antibody, β-tubulin (mouse, monoclonal, 1:1000, Sigma-Aldrich), overnight in PBS, samples were permeabilized and blocked in 0.1% Triton X-100 with 10% normal goat serum (NGF, Vector Laboratories Inc., Burlingame, CA, USA) for 1 h. The secondary antibody, goat anti-mouse Alexa fluor 488 (1:200, Molecular Probes, Eugene, Oregon), in PBS, was incubated for 1 h at RT.

Dako fluorescent mounting media were used to mount the cells. A Zeiss LSM800 confocal laser microscope system was used to capture the images. According to previously stated criteria, the differentiated and undifferentiated cells were manually counted [26]. The differentiated cells were identified by the presence of multiple neurites, at least one of which had a length that was at least twice as long as the smallest cell diameter. Using ImageJ software 1.51k, the lengths of the neurites were also measured.

#### 2.2.6. Neurite Outgrowth Assay Using Ex Vivo Models

#####  Dorsal Root Ganglia (DRG) Dissection and Cultures

In this investigation, adult female Wistar rats (Envigo, Udine, Italy) weighing 190–220 g were employed. All procedures were carried out in compliance with the requirements of Directive EU/2010/63 of the European Union. Before the study started, it also received approval from the University of Turin’s Ethics Experimental Committee (Ministry of Health project number 864/2016). Given that humans are the endpoints for animal suffering and distress, adequate efforts were made to reduce pain and discomfort. Lethal injections of the anesthetic mixture, including Tiletamine + Zolazepam (Zoletil) i.m. (3 mg/kg), were used to kill the animals. To acquire ventral access to the spinal cord, the vertebral body was cut out surgically and the vertebral column was dissected. The DRGs from all spinal levels were collected. Each ganglion was divided in half after the DRG was harvested, and the connective tissue capsule around the ganglia was removed. Before seeding the explants, a 50 µL drop of Thermo Fisher Scientific’s Geltrex Matrigel in F12 medium (50 percent *v*/*v*) was applied to the substrate [27].

Serum-free culture media containing NGF (50 ng/mL, Invitrogen) was added following a 2 h incubation period at 37 °C. At 37 °C and 5% CO_2_, explants were kept in a predetermined serum-free medium (SFM) or in SFM containing the degradation products with Si isolated from the porous hybrids.

##### Immunofluorescence

Explants were fixed in 4% PAF for 15 min at room temperature after 3 days of culture. Specimens were incubated overnight in a solution comprising anti-peripherin (polyclonal rabbit, 1:1000, Chemicon International Inc.) and anti-neurofilament −200 kDa (monoclonal mouse, 1:200, Thermo Fisher Scientific) primary antibodies. After PBS washing, sections were subjected to double immunolabeling by being incubated for 1 h with a solution containing the secondary antibodies: anti-mouse IgG Alexa Fluor 488-conjugated (Molecular Probes) and anti-rabbit IgG Cy3 (Jackson ImmunoResearch Laboratories Inc., West Grove, PA, USA). Following this, explants were mounted using a Dako fluorescent mounting medium.

##### Quantification of Neurite Outgrowth

Six samples from each experimental group were analyzed. An LSM 510 confocal laser microscopy device (Zeiss, Jena, Germany) was used to make confocal Z-stacks on each sample, and various pictures were used to reconstruct the whole ganglia [28]. Using Neurite J (an ImageJ plugin), the parameters for neurite extension and axonal sprouting were assessed in order to assess the neurite outgrowth of the DRG explants.

### 2.3. Statistical Analysis

Technical and biological triplicates of the experiments on cells were carried out, and a DRG morphological examination was performed on six samples for each experimental group. The results for cell proliferation and morphology were examined using one-way analysis of variance (ANOVA) followed by Bonferroni tests and displayed as means with standard deviations (SD).

## 3. Results and Discussion

### 3.1. Degradation Products Containing Si in the Extractions

**Figure 1:**
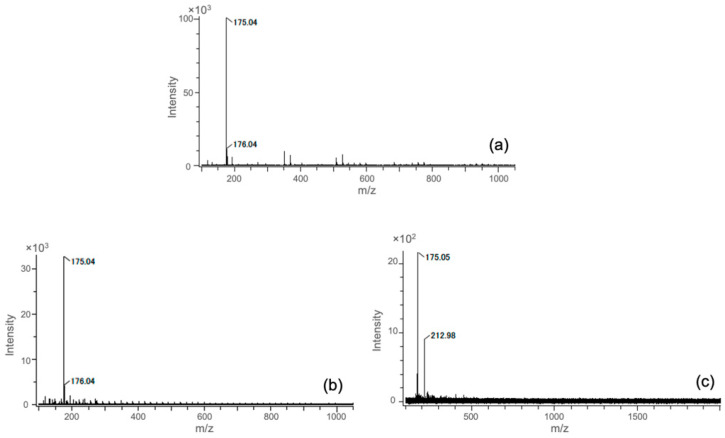
ESI negative TOF-MS spectra of (**a**) hydrolyzed GPTMS in an HOAc aqueous solution, (**b**) ChG05 extraction solution, and (**c**) ChG10 extraction solution.

**Figure 2:**
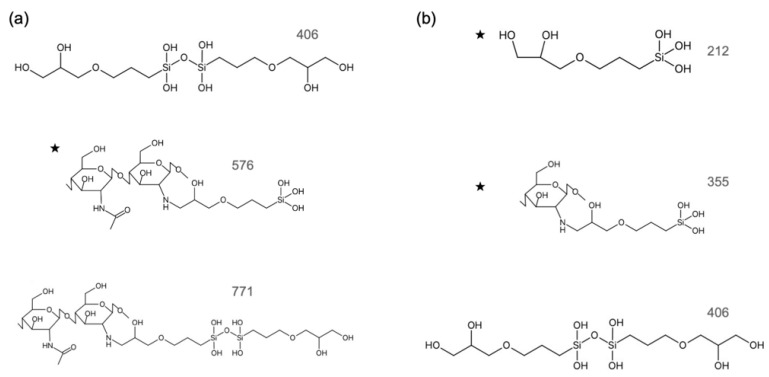
The structures and molecular weights of the degradation products with Si dissolved from the hybrids in the extractions, estimated using TOF-MS spectra. (**a**) ChG05. (**b**) ChG10. ★ Main degradation products containing Si in the extraction.

The concentration of Si ions in the extractions depended on the starting composition of the hybrids. According to the ICP results, the concentrations of Si ions in the culture medium were chosen as 0.05 mM and 0.10 mM from ChG05, and 0.10 mM and 0.50 mM from ChG10. The degradation of the chitosan–GPTMS hybrids depended on the amount of GPTMS because the epoxy groups of GPTMS crosslinked with the amino groups of chitosan and methoxy silane groups of GPTMS formed siloxane networks [15,16]. The degradation of the hybrids occurred by cleavage of the *β*(1 → 4) glucosamine bridge or –NH–C– between GPTMS and chitosan. The free amino groups in both extractions were not detected using the ninhydrin method. Therefore, the amino groups in both extractions bonded with GPTMS. Figure 1 shows the ESI negative TOF-MS spectra of GPTMS/HOAc and the extractions from ChG05 and ChG10. The estimated structures of the degradation products with Si extracted from the hybrids and the molecular weights are shown in Figure S1. The methoxy groups of GPTMS were completely hydrolyzed in the extractions. Peaks near *m*/*z* 175 were detected in all spectra and can be attributed to the monomeric compounds from the ring-opened and hydrolyzed GPTMS, as shown in Figure S1a,c [20]. A peak at *m*/*z* 212.98 was detected in only the ChG10 spectra, as shown in Figure 1c, responding to the monomeric compound of the hydrolyzed GPTMS, as shown in Figure S1c. A peak at *m*/*z* 161 from the glucosamine unit was detected in both extractions; alternatively, a peak at *m*/*z* 221 from the *N*-acetyl glucosamine unit was detected only in the ChG05 extraction (Figures S2 and S3, Tables S1 and S2). A peak near *m*/*z* 400, attributed to the dimer of the condensed GPTMS, was detected in both extractions (Figure S1b,c). Figure 2 shows the estimated structures and molecular weights of the degradation products with Si in the extractions using TOF-MS spectra. The Si–O–Si condensation increased as the amount of GPTMS increased [15,16]. As a result, it was easier to condense between silanol groups (–Si–OH) and break the *β*(1 → 4) glucosamine bridge of the ChG10 hybrids compared to the Si–O–Si bond. In the case of the extraction from ChG05, an *N*-acetyl glucosamine unit was detected, indicating cleavage at the *β*(1 → 4) glucosamine bridge next to the chitin unit. The degradation products with Si in the extractions of ChG05, including both chitin and chitosan units, were larger (Figure 2a) compared to those of ChG10.

### 3.2. RT4-D6P2T Cytocompatibility

**Figure 3:**
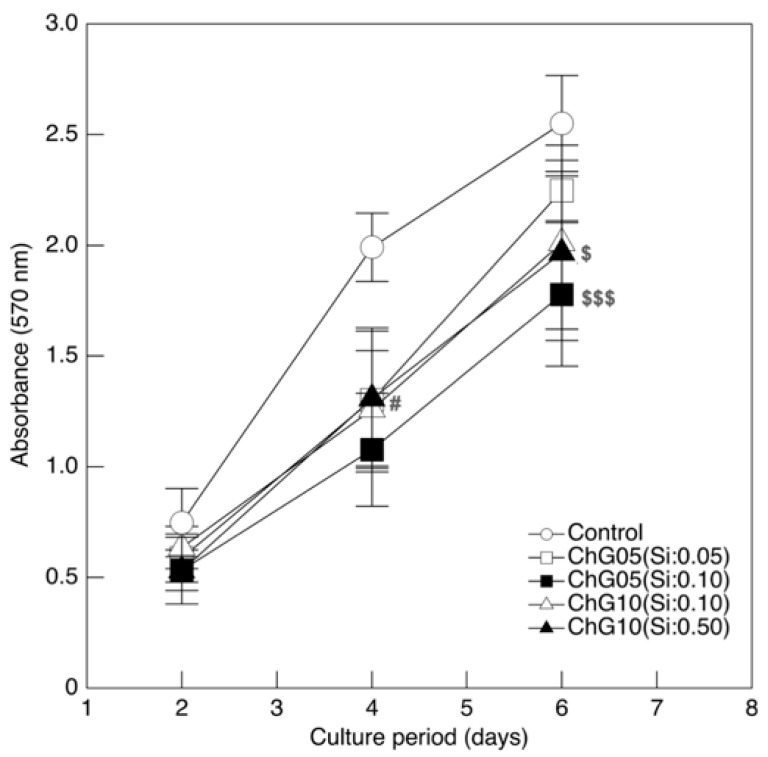
Cell proliferation of RT4-D6P2T cells cultured in control conditions or in a medium with extractions. Differences at each time point between all tested groups and control are reported. ^$^ *p* < 0.05, ^$$$^ *p* < 0.001 significantly different from ChG05(Si:0.05), ^#^ *p* < 0.05 significantly different from ChG05(Si:0.10).

**Figure 4:**
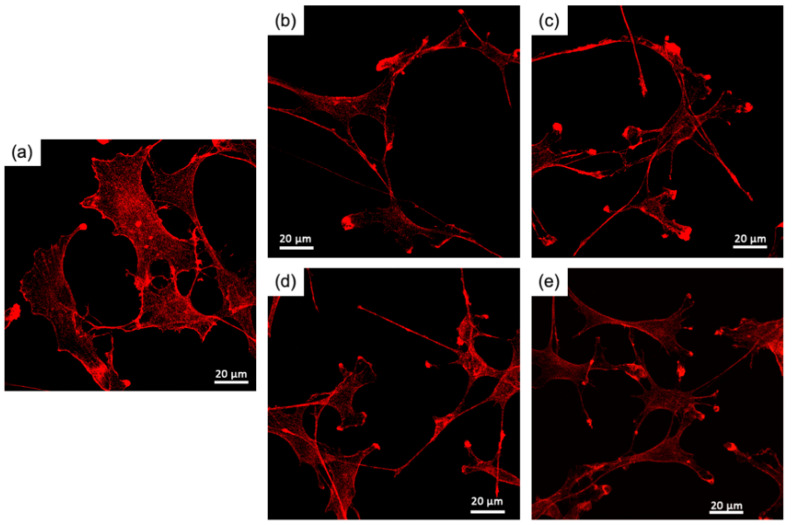
Fluorescence images after phalloidin staining (red) of RT4-D6P2T cells after 2 days of culture on glass slides in control conditions or in a medium with extractions: (**a**) control, (**b**) ChG05(Si:0.05), (**c**) ChG05(Si:0.10), (**d**) ChG10(Si:0.10), (**e**) ChG10(Si:0.50).

Figure 3 shows the RT4-D6P2T cell proliferation in the medium with the extraction products. Cells were shown to maintain proliferative behavior under all conditions tested, however, the value of MTT was lower than in the control medium throughout the culture period, indicating that cell proliferation was inhibited by the degradation products with Si in the extractions, despite the extraction conditions. In particular, cell proliferation was significantly lower compared to the control at 4 d and 6 d for the group represented by ChG05(Si:0.10). The Si ion concentration affected cell proliferation in the ChG05 series but not in the ChG10 series. ChG10(Si:0.50) had the highest concentration of Si ions among the experimental groups; however, the value of MTT was greater compared to ChG05(Si:0.10). Therefore, cell proliferation was inhibited by the concentration of the degradation products with Si and their molecular structures or molecular weights. The degradation products with Si from the chitosan–GPTMS hybrids were not taken up by the cells because of their larger molecular weights [20]. The molecular weights of the degradation products extracted from the porous hybrids were lower compared to those from the solid membranes, indicating that the decrease in cell proliferation by the degradation products from the porous hybrids interacted with cell surfaces or biological molecules in the culture medium. Figure 4 and Figure S2 show fluorescence images of RT4-D6P2T cells cultured on glass slides in the control or medium with the extraction at 2 d and 4 d and stained with phalloidin to evaluate the organization of the actin cytoskeleton. The cells in the control were spread, whereas the cells in the medium with the extraction had an elongated morphology.

**Figure 5:**
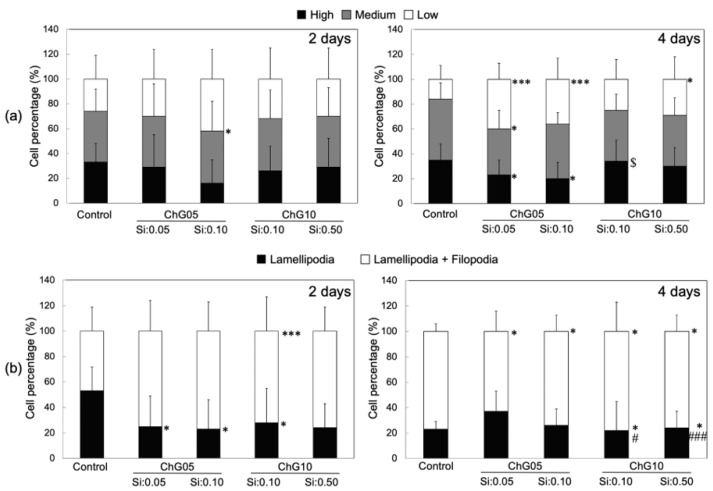
Percentages of RT4-D6P2T cells (**a**) with low, medium, or high actin cytoskeleton organization, and (**b**) characterized by lamellipodia and filopodia organization protrusions. Cells were cultured for 2 and 4 days on glass slides in control conditions or in a medium with extractions. Differences at each time point between groups are reported: * *p* < 0.05, *** *p* < 0.001 significantly different from control. ^#^ *p* < 0.05, ^###^ *p* < 0.001 significantly different from ChG05(Si:0.05). ^$^ *p* < 0.05 significantly different from ChG05(Si:0.10).

**Figure 6:**
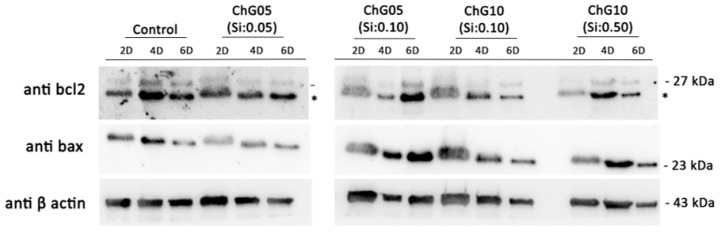
Western blot analysis for Bcl2 and Bax protein expression of RT4-D6P2T cells cultured on glass slides in control conditions or in medium with extractions. Anti-β-actin antibodies were used as the control. * is unspecific bands. 27 kDa, 23kDa, and 43 kDa are corresponding to anti-bcl2, anti bax, and anti β actin, respectively. The original figures were provided in Figures S4–S6.

The percentage of elongated cells increased at 4 d and was greater compared to that of the control, particularly in the medium from the ChG05 series, as shown in Figure 5a. The staining carried out made it possible to distinguish the cytoskeleton organization between a prevalence of filopodia or lamellipodia in RT4-D6P2T cells. The former was highly organized, with tightly crosslinked long bundles associated with cell motility and migration, whereas the latter was represented by a thin sheet-like branched network of actin filaments, most represented during cell adhesion. Figure 5b shows the percentages of cells characterized by lamellipodia and filopodia organization. At 2 d, the cells cultured in the medium with extractions had more filopodia than the control. The percentage of filopodia increased in the control and decreased in the case of the ChG05(Si:0.05) at 4 d. The ChG10 series showed a slightly higher percentage of filopodia than the control at 4 d. The larger-molecular-weight degradation products with Si (Figure 2a) in the ChG05 series were adsorbed on the cell surfaces [20] and then filopodia formation was inhibited. To further evaluate the biocompatibility of the extraction products, two proteins involved in the mechanisms of cellular apoptosis were evaluated, anti-apoptotic Bcl2 and pro-apoptotic Bax (Figure 6). The only treatment in which the expression of pro-apoptotic Bax increased was ChG05(Si: 0.10). Nevertheless, the cells cultured in the presence of the four different extraction products exhibited an initial decrease in anti-apoptotic Bcl2, and in all cases, the protein increased progressively at 4 d and 6 d of culture, indicating a cellular adaptation to the extraction products and activation of the anti-apoptotic signal. RT4-D6P2T cells have been shown to respond well to different culture conditions, maintaining good proliferative capacity, structural organization, and protein expression linked to cell survival. The only treatment in which the cells deviated significantly from the control conditions was ChG05(Si:0.10), in which proliferation and actin cytoskeleton organization decreased with a concomitant upregulation of the pro-apoptotic Bax protein.

### 3.3. NSC-34 Differentiation

**Figure 7:**
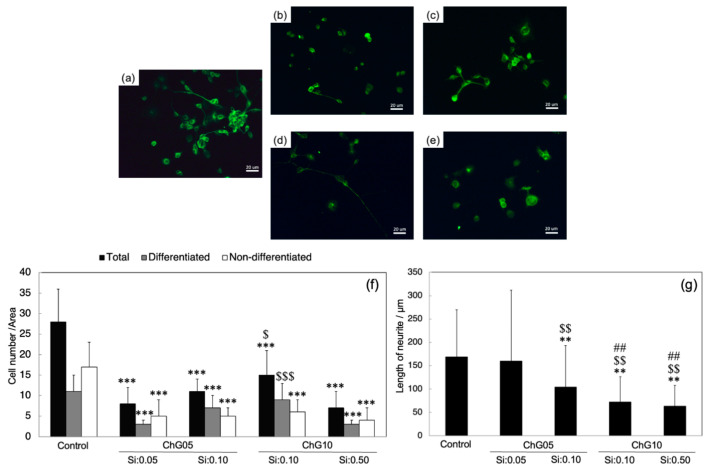
Fluorescence images (**a**–**e**) after β-tubulin staining (green), and graphs (**f**,**g**) representing the number of neurites and the neurite length of NSC-34 cells after 5 days of culture on glass slides in control conditions or in a medium with extractions: (**a**) control, (**b**) ChG05(Si:0.05), (**c**) ChG05(Si:0.10), (**d**) ChG10(Si:0.10), (**e**) ChG10(Si:0.50), (**f**) number of neurites, and (**g**) neurite length. Differences at each time point between groups are reported: ** *p* < 0.01, *** *p* < 0.001 significantly different from the respective control. ^$^ *p* < 0.05, ^$$^ *p* < 0.01, ^$$$^ *p* < 0.001 significantly different from ChG05(Si:0.05). ^##^ *p* < 0.01 significantly different from ChG05(Si:0.10).

NSC-34 is a hybrid cell line produced by the fusion of motor neurons from the spinal cord of mouse embryos and N18TG2 neuroblastoma cells [29]. These cells exhibit properties of motor neurons when subjected to differentiation and maturation protocols. Figure 7a–e show the images of NSC-34 cells cultured at 5 d on glass slides in a differentiation medium with the extractions, and Figure 7f,g show the evaluations of the numbers of differentiated and non-differentiated cells, as well as the neurite length. The number of cells cultured in the medium with extractions was less than the control, indicating that the degradation products with Si reduced NSC-34 cell survival. The differentiation ratios of the control, ChG05(Si:0.05), ChG05(Si:0.10), ChG10(Si:0.10), and ChG10(Si:0.50) were 40%, 33%, 64%, 60%, and 43%, respectively. Specifically, neuronal differentiation was enhanced by the degradation products with Si, regardless of the molecular weight. Regarding the neurite length, ChG05(Si:0.05) was similar to the control, and others showed a significantly reduced length. Additionally, the neurites of the ChG10 series were shorter compared to ChG05(Si:0.10). In previous studies, the nerve regeneration with the chitosan–GPTMS porous hybrids (ChG05) was found to recover motor function [17]. The differentiation analysis of NSC-34 cells indicates that the degradation products with Si from the porous hybrids can promote the induction of the motor neuron phenotype.

### 3.4. Neurite Outgrowth Assay

**Figure 8:**
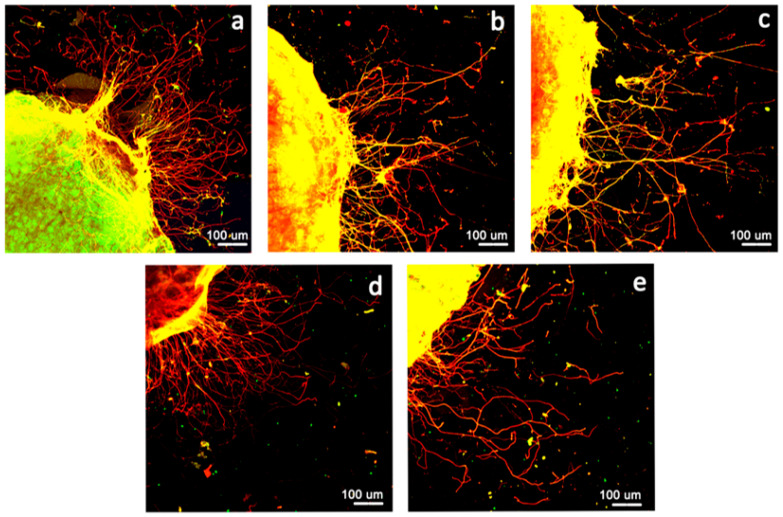

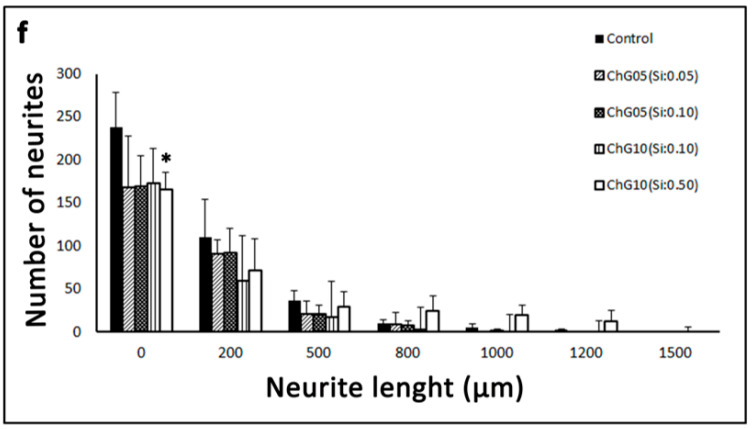
Representative immunostaining images (**a**–**e**) of axonal outgrowth (neurofilament in green and peripherin in red) and graph representing the number of neurites and neurite length of adult DRG explants cultured for 3 days in control conditions or in a medium with extractions: (**a**) control, (**b**) ChG05(Si:0.05), (**c**) ChG05(Si:0.10), (**d**) ChG10(Si:0.10), (**e**) ChG10(Si:0.50), and (**f**) the number of neurites and neurite length. * *p* < 0.05 significantly different from the respective control.

Figure 8a–e show the axonal outgrowth from the DRG explants after 3 d of culture on a Matrigel-coated coverslip in a medium with the extractions. The use of organotipic cultures allowed us to obtain a multicellular ex vivo model that preserves both the cytoarchitecture of the tissue and the interactions among cells. In order to better evaluate neurite outgrowth in a control medium or with extractions, neurites were marked with two axonal antibodies: anti-NF-200 kDa antibody (green) and anti-peripherin (red). Neurite outgrowth was observed in the medium with the extraction products, similar to the control conditions. Figure 8f shows the number of neurites and neurite length. ChG10(Si:0.50) slightly increased the neurite outgrowth extensions. The polycationic chitosan enhanced the neurite extension of the DRG [30]. The degradation products with Si were not positively charged by the chitosan units. Therefore, the units with Si affected the neurite outgrowth. Our previous results regarding nerve regeneration with a chitosan–GPTMS solid membrane and porous scaffold [17] showed different nerve regeneration. This ex vivo study suggests that the porous structure provides favorable conditions for neurites’ elongation.

## 4. Conclusions

The molecular structures of the degradation products with Si from porous chitosan–GPTMS hybrids were investigated using TOF-MS spectrometry. The porous hybrids were degraded by cleavage at the *β*(1 → 4) glucosamine bridge, not between chitosan and GPTMS. The RT4-D6P2T proliferation results indicate that the larger-molecular-weight degradation products with Si had filopodia formation, compatible with a migrating glial phenotype, and expressed more of the pro-apoptotic Bax protein. The differentiation of NSC-34 cells and DRG neurite extension were promoted by the degradation products with Si extracted from the porous hybrids. Our results suggest that lower-molecular-weight products with Si are useful for nerve regeneration, and it is important to understand the impact of products dissolved from or degraded by the materials on cell responses.

## Figures and Tables

**Table 1:** Antibodies used for Western blot analysis.

Antibodies for Western Blot Analysis				
	Code	Dilution	Host	Source
**Primary antibodies**				
Bcl2	sc-492	1:300	rabbit	Santa Cruz Biotechnology Inc
BAX	sc-23959	1:500	mouse	Santa Cruz Biotechnology Inc
β-actin	A5316	1:4000	mouse	Sigma
**Secondary antibodies**				
HRP-conjugated anti-rabbit	7074	1:15,000	goat	Cell signaling
HRP-conjugated anti-mouse	7076	1:15,000	goat	Cell signaling

## Data Availability

The authors confirm that the data supporting the findings of this study are available within the article and its Supplementary Materials.

## Supplementary Materials

The following supporting information can be downloaded at: https://www.mdpi.com/article/10.3390/polym15153272/s1, Figure S1. The structure and molecular weight of the degradation products with Si dissolved from chitosan–GPTMS hybrids; (a) chitin/chitosan and GPTMS monomer, (b) chitosan and GPTMS dimers, and (c) GPTMS dimers. Figure S2. ESI negative TOF-MS original data of the ChG05 extraction; Figure S3. ESI negative TOF-MS original data of the ChG10 extraction; Figure S4. Original data of anti-bcl2; Figure S5. Original data of anti-Bax (chemiluminescence); Figure S6. Original data of β-actin (chemiluminescence). Table S1. ESI negative TOF-MS data of all peaks for the ChG05 extraction; Table S2. ESI negative TOF-MS data of all peaks for the ChG10 extraction.
