# Correction: Shirosaki et al. The Impact of the Molecular Weight of Degradation Products with Silicon from Porous Chitosan–Siloxane Hybrids on Neuronal Cell Behavior. *Polymers* 2023, *15*, 3272

**DOI:** 10.3390/polym18162019

**Published:** 2026-08-20

**Authors:** Yuki Shirosaki, Federica Fregnan, Luisa Muratori, Saki Yasutomi, Stefano Geuna, Stefania Raimondo

**Affiliations:** 1 Faculty of Engineering, Kyushu Institute of Technology, 1-1 Sensui-cho, Tobata-ku, Kitakyushu 804-8550, Japan; s-yasutomi@che.kyutech.ac.jp; 2 Department of Clinical and Biological Sciences and Cavalieri Ottolenghi Neuroscience Institute, University of Turin, Regione Gonzole 10, 10043 Orbassano, Italy; federica.fregnan@unito.it (F.F.); luisa.muratori@unito.it (L.M.); stefano.geuna@unito.it (S.G.); stefania.raimondo@unito.it (S.R.)

## Error in Figure

In the original publication [1], there was a mistake in Figure 8. Specifically, the image shown in Figure 8e did not correspond to the indicated experimental group ChG10 (Si:0.50) due to an error during figure preparation.

The corrected Figure 8 appears below, in which Figure 8e has been replaced with the appropriate image. The same image was also included in the graphical abstract, and the graphical abstract has therefore been revised accordingly to replace the incorrect image with the appropriate one.

The authors have carefully re-examined the original data and confirm that the quantitative analyses associated with Figure 8 were performed using the correct dataset. 

The authors state that the scientific conclusions are unaffected. This correction was approved by the Academic Editor. The original publication has also been updated.

## Figures and Tables

**Figure 8:**
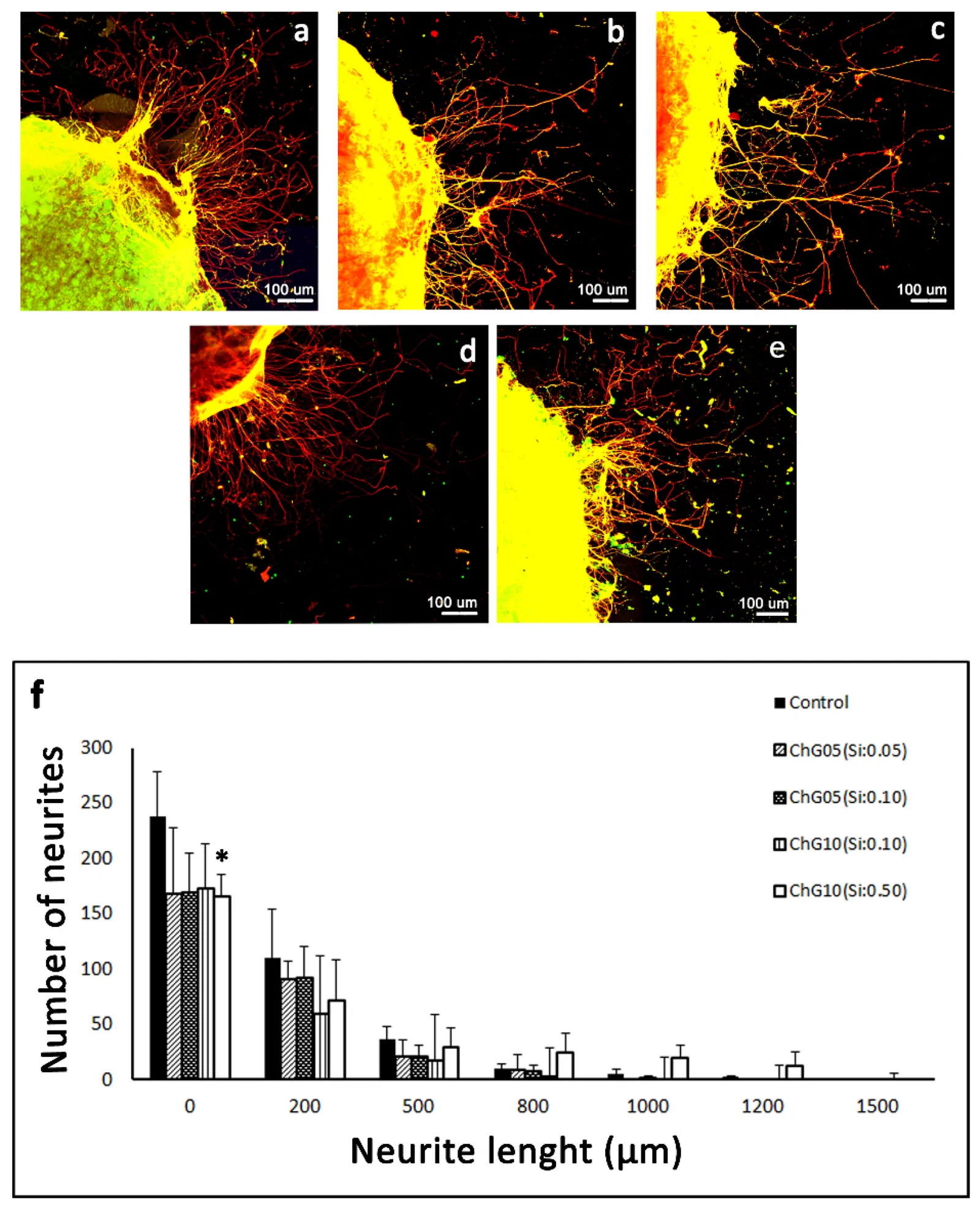
Representative immunostaining images (**a**–**e**) of axonal outgrowth (neurofilament in green and peripherin in red) and graph representing the number of neurites and neurite length of adult DRG explants cultured for 3 days in control conditions or in a medium with extractions: (**a**) control, (**b**) ChG05(Si:0.05), (**c**) ChG05(Si:0.10), (**d**) ChG10(Si:0.10), (**e**) ChG10(Si:0.50), and (**f**) the number of neurites and neurite length. * *p* < 0.05 significantly different from the respective control.
