# Supplementary material for: The Impact of the Molecular Weight of Degradation Products with Silicon from Porous Chitosan–Siloxane Hybrids on Neuronal Cell Behavior
Source: Polymers (Basel). 2023 Aug 1;15(15):3272. doi: 10.3390/polym15153272 (PMC10422348; doi:10.3390/polym15153272)
Supplement: Supplementary file 1 [file polymers-15-03272-s001.zip › polymers-2488453-supplementary.pdf]

Supplementary material

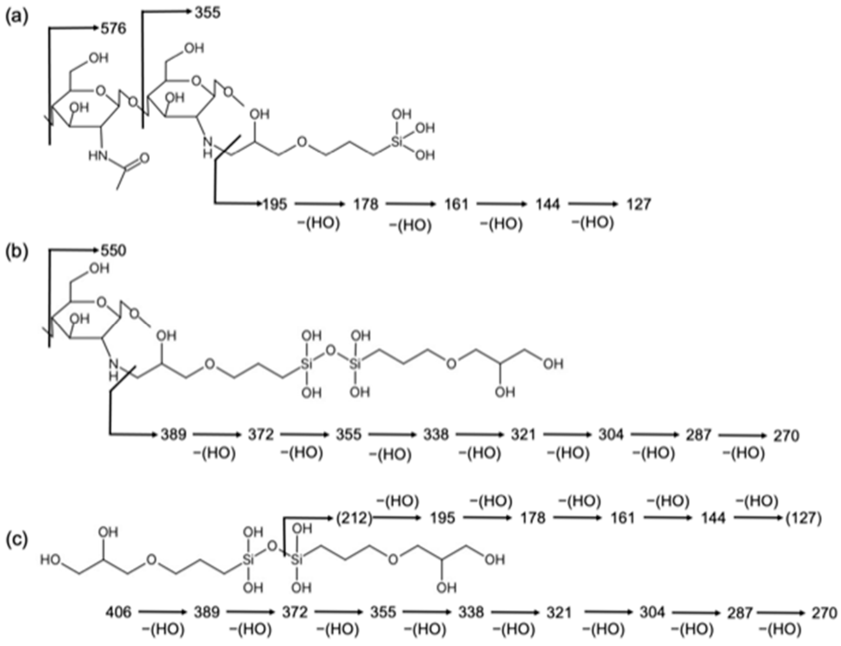

Figure S1. The structure and molecular weight of the degradation products with Si dissolved from chitosan–GPTMS hybrids; (a) chitin/chitosan and GPTMS monomer, (b) chitosan and GPTMS dimers, and (c) GPTMS dimers.

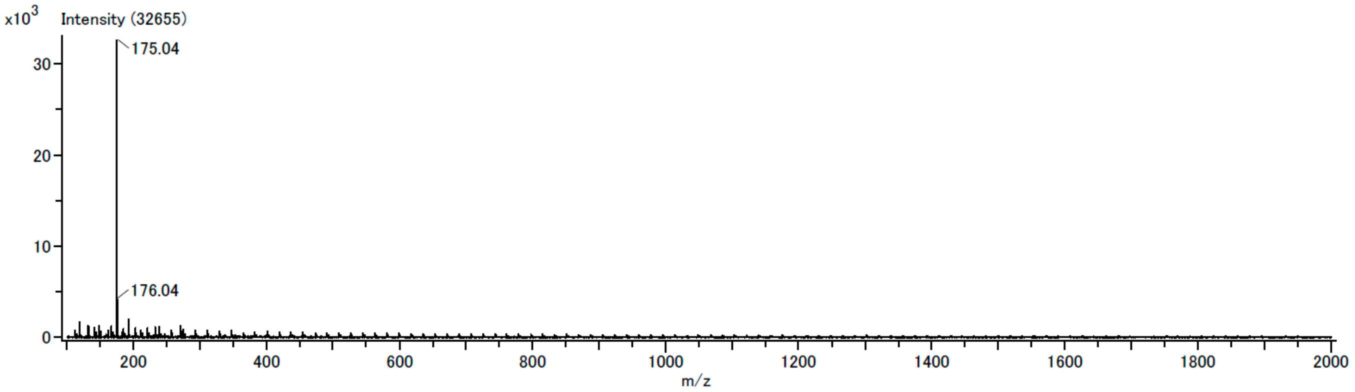

Figure S2. ESI negative TOF-MS data of the ChG05 extraction.

Peak#	m/z	Area	Area[% Max.]	Height	Height[% Max.]	Description
1	113.04	1226.05	2.37	808.91	2.48	
2	119.01	2933.93	5.67	1668.23	5.11	
3	131.05	1734.28	3.35	1270.41	3.89	
4	132.99	1864.20	3.60	1220.68	3.74	
5	133.03	978.06	1.89	613.82	1.88	
6	141.02	1604.05	3.10	1020.82	3.13	
7	145.03	825.13	1.60	530.14	1.62	
8	149.07	1730.40	3.35	1271.68	3.89	
9	151.05	993.63	1.92	619.45	1.90	
10	162.96	1063.67	2.06	747.00	2.29	
11	167.07	1771.99	3.43	1187.32	3.64	
12	169.06	911.32	1.76	524.14	1.61	
13	175.04	51723.16	100.00	32655.09	100.00	
14	176.04	6788.16	13.12	4101.23	12.56	
15	177.04	2406.36	4.65	1448.59	4.44	
16	183.01	994.66	1.92	578.59	1.77	
17	185.08	1441.05	2.79	888.82	2.72	
18	193.05	3009.68	5.82	1973.59	6.04	
19	203.10	1515.69	2.93	990.73	3.03	
20	211.06	1298.29	2.51	721.32	2.21	

Table S1. ESI negative TOF-MS data of all peaks for the ChG05 extraction.

Peak#	m/z	Area	Area[% Max.]	Height	Height[% Max.]	Description
21	221.11	2091.93	4.04	977.18	2.99	
22	233.04	1981.72	3.83	1095.23	3.35	
23	239.11	1966.72	3.80	1165.09	3.57	
24	257.05	1312.29	2.54	766.23	2.35	
25	257.13	1302.20	2.52	752.86	2.31	
26	271.08	1955.32	3.78	1277.95	3.91	
27	273.06	1382.51	2.67	618.27	1.89	
28	275.14	1571.13	3.04	825.59	2.53	
29	293.15	1291.59	2.50	722.14	2.21	
30	311.16	1248.22	2.41	756.82	2.32	
31	329.18	1163.22	2.25	629.55	1.93	
32	347.18	1281.39	2.48	807.05	2.47	
33	365.20	857.60	1.66	495.14	1.52	
34	383.20	1145.59	2.21	564.73	1.73	
35	401.21	1228.14	2.37	604.14	1.85	
36	419.23	1218.82	2.36	578.55	1.77	
37	437.24	1034.23	2.00	541.64	1.66	
38	455.24	1042.97	2.02	529.09	1.62	

Table S1(continue). ESI negative TOF-MS data of all peaks for the ChG05 extraction.

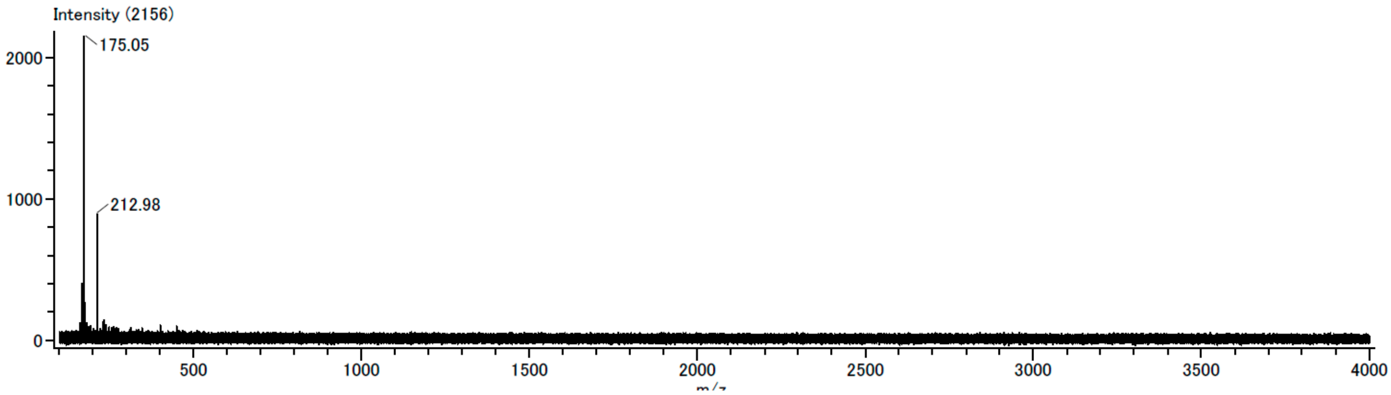

Figure S3. ESI negative TOF-MS data of the ChG10 extraction

Peak#	m/z	Area	Area[% Max.]	Height	Height[% Max.]	Description
1	162.96	225.91	6.57	122.59	5.69	
2	168.99	652.58	18.98	402.00	18.65	
3	175.05	3437.69	100.00	2155.90	100.00	
4	176.04	520.78	15.15	266.79	12.38	
5	177.04	250.79	7.30	102.54	4.76	
6	183.01	305.34	8.88	122.31	5.67	
7	193.06	185.33	5.39	101.49	4.71	
8	212.98	1578.82	45.93	889.72	41.27	
9	230.99	306.01	8.90	132.03	6.12	
10	233.04	298.78	8.69	144.62	6.71	
11	239.12	207.03	6.02	108.44	5.03	
12	400.86	210.18	6.11	106.77	4.95	
13	448.96	172.11	5.01	100.10	4.64	

Table S2. ESI negative TOF-MS data of all peaks for the ChG10 extraction.

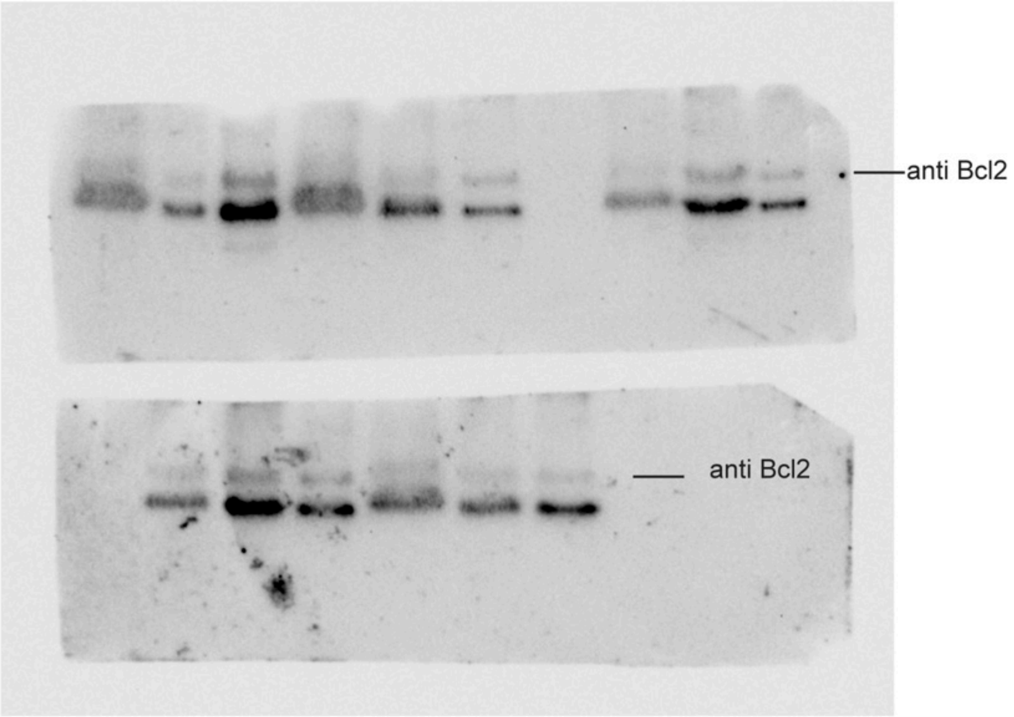

Figure S4. Original data of anti bcl2.

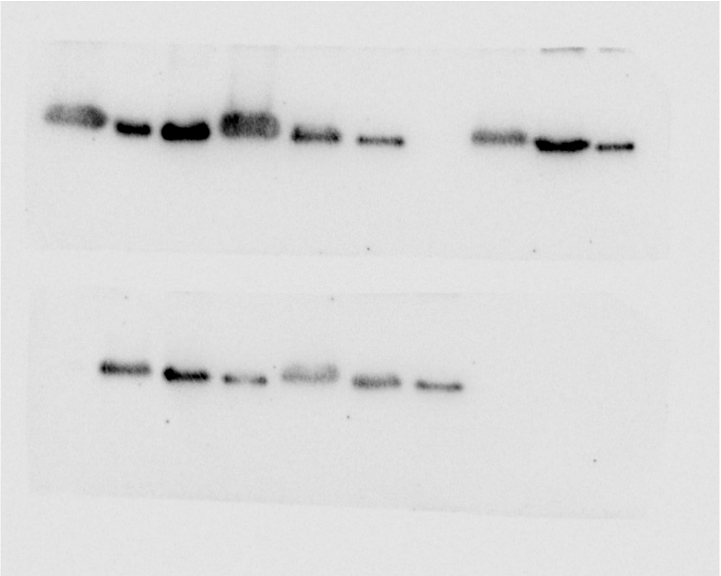

Figure S5. Original data of anti bax (Chemiluminescence).

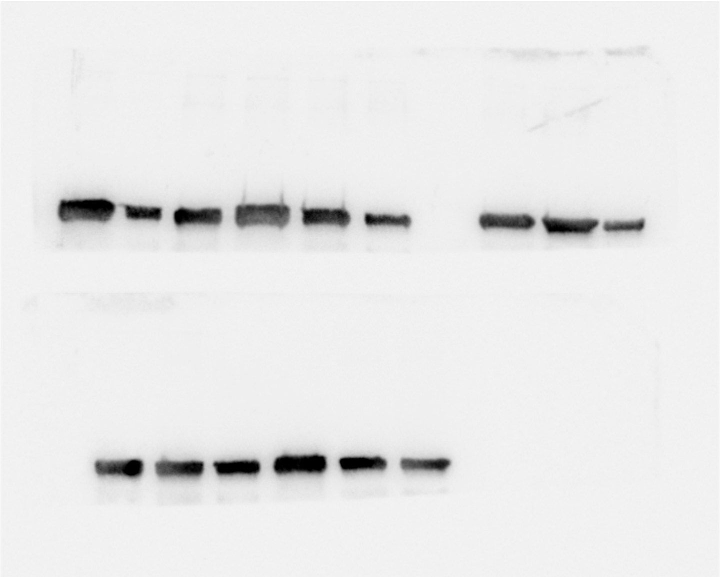

Figure S6. Original data of β-actin (Chemiluminescence).
